# Genetic heterogeneity in autosomal recessive hearing loss: a survey of Brazilian families

**DOI:** 10.3389/fgene.2024.1409306

**Published:** 2024-10-21

**Authors:** Larissa Nascimento Antunes, Alex Marcel Moreira Dias, Beatriz Cetalle Schiavo, Beatriz C. A. Mendes, Debora Romeo Bertola, Karina Lezirovitz, Regina Célia Mingroni-Netto

**Affiliations:** ^1^ Centro de Pesquisas sobre o Genoma Humano e Células-Tronco, Departamento de Genética e Biologia Evolutiva, Instituto de Biociências, Universidade de São Paulo, São Paulo, Brazil; ^2^ Divisão de Educação e Reabilitação dos Distúrbios da Comunicação da Pontifícia Universidade Católica de São Paulo, São Paulo, Brazil; ^3^ Unidade de Genética do Instituto da Criança, Faculdade de Medicina, Universidade de São Paulo, São Paulo, Brazil; ^4^ Laboratório de Otorrinolaringologia/LIM32 - Hospital das Clínicas, Faculdade de Medicina, Universidade de São Paulo, São Paulo, Brazil

**Keywords:** hearing loss, autosomal recessive, next-generation sequencing, hereditary deafness, molecular diagnosis

## Abstract

**Introduction:**

Hearing loss is a frequent sensory impairment type in humans, with about 50% of prelingual cases being attributed to genetic factors. Autosomal recessive hearing loss (ARHL) exhibits great locus heterogeneity and is responsible for 70%–80% of hereditary nonsyndromic cases.

**Methods:**

A total of 90 unrelated Brazilian individuals were selected for having hearing loss of presumably autosomal recessive inheritance, either born from consanguineous marriages or belonging to families with two or more affected individuals in the sibship and most cases were of normal hearing parents. In all cases, common pathogenic variants in *GJB2* (c.35delG), *GJB6* [del(GJB6-D13S1830) and del(GJB6-D13S1854)] and *MT-RNR1* (m.1555A>G) were discarded and most were previously assessed by complete Sanger sequencing of *GJB2*. Their genetic material was analyzed through next-generation sequencing, targeting 99 hearing loss-related genes and/or whole exome sequencing.

**Results:**

In 32 of the 90 probands (36,7%) causative variants were identified, with autosomal recessive inheritance confirmed in all, except for two cases due to dominant variants (*SIX1* and *P2RX2*). Thirty-nine different causative variants were found in 24 different known hearing loss-associated genes, among which 10 variants are novel, indicating wide genetic heterogeneity in the sample, after exclusion of common pathogenic variants. Despite the genetic heterogeneity, some genes showed greater contribution: *GJB2*, *CDH23*, *MYO15A*, *OTOF*, and *USH2A*.

**Conclusion:**

The present results confirmed that next-generation sequencing is an effective tool for identifying causative variants in autosomal recessive hearing loss. To our knowledge, this is the first report of next-generation sequencing being applied to a large cohort of pedigrees with presumable autosomal recessive hearing loss in Brazil and South America.

## 1 Introduction

Hearing loss is a frequent type of sensory impairment in humans. In developed countries, around 50% of cases are due to genetic alterations (Morton and Nance, 2006), and 70% are nonsyndromic. Estimates of the prevalence of hearing loss in Brazil are scarce. Brazil has older newborn hearing screening programs, when compared to other countries in Latin America, but the law determining that Universal Neonatal Hearing Screening is mandatory for all children was approved only in 2010 (Lezirovitz and Mingroni-Netto, 2022). Even after that, coverage of the screening is incomplete and heterogeneous in different regions of the country, making it difficult to obtain precise estimates of the prevalence of hearing loss among newborns (Dias et al., 2024). The Health Ministry of Brazil presented as 24% the coverage index of newborn hearing screening. Absence of a public policy, national coordination and a national database for newborn screening makes it difficult to estimate how many children are submitted to the hearing screening in the proper time (Neumann et al., 2020).

Hereditary hearing loss displays high heterogeneity in inheritance patterns, locus, alleles, and phenotypes (Shearer et al., 2020). Autosomal recessive hearing loss is responsible for 70%–80% of hereditary nonsyndromic cases (Shearer et al., 2020) and exhibits impressive locus heterogeneity. Causative genetic variants in 83 genes have been related to nonsyndromic autosomal recessive hearing loss (Walls et al., 2024). Syndromic hearing loss represents 30% of genetic cases and is equally genetically heterogeneous.

Pathogenic variants within the DFNB1 locus (13q11-12), which contains the genes *GJB2* and *GJB6*, are estimated to explain near 50% of all cases of autosomal recessive hearing loss in many different countries and regions. (Guilford et al., 1994; Kenneson et al., 2002; Smith and Jones, 2016). The *GJB2* (Gap Junction Beta 2) gene (OMIM# 121011) encodes connexin (Cx) 26 (Cx26), a member of the large family of gap junction proteins (Nielsen et al., 2012). Among all known *GJB2* pathogenic variants, c.35del:p.(Gly12ValfsTer2), NC_000013.11, OMIM *121011.005) is the most frequent, corresponding to near 75% of all reported alleles with a DFNB1 pathogenic variant in European or European derived populations.

The *GJB6* gene encoding connexin 30 (Cx30) is adjacent to the *GJB2* gene in the DFNB1 locus. Large deletions encompassing the *GJB6* gene or neighbouring regions have been reported either in homozygosis or in trans with a single recessive mutation in the *GJB2* gene determining hearing loss. These *GJB6*-related deletions act as recessive alleles with *GJB2* variants (del Castillo et al., 2002; del Castillo et al., 2005).

In Brazil, 10%–15% of nonsyndromic cases were found to be caused by variants in *GJB2* and *GJB6* related deletions (Batissoco et al., 2009; Batissoco et al., 2022). Due to the high locus heterogeneity, it has been difficult, especially in developing countries, to screen for other genes that are related to autosomal recessive hearing loss. However, some studies from the United States, Belgium, Northern Europe, and East Asia indicated that pathogenic variants in *SLC26A4*, *MYO15A*, *OTOF*, and *CDH23* are probably the most common causes of autosomal recessive hearing loss, after the exclusion of variants in the DFNB1 locus (Hilgert et al., 2009; Duman and Tekin, 2012).

For many years, genetic testing for hearing loss was not a trivial issue. The development of next-generation sequencing (NGS) brought a new perspective to it (Shearer and Smith, 2012). This technique allows the simultaneous sequencing of several genes. Its use in genetically heterogeneous conditions, such as hearing loss, is effective in molecular investigation, and it is recommended for all subjects after screening for variants in the DFNB1 locus, with normal results (Alford et al., 2014; Wright, Fitzpatrick, Firth, 2018). However, it is still not affordable for the majority of the population in developing countries. Thus, its application to big research cohorts may help prove its effectiveness for other neglected populations.

This study aimed to use NGS to contribute to the understanding of the genetic heterogeneity in autosomal recessive hearing loss, in a Brazilian casuistic selected for having hearing loss of presumptive autosomal recessive inheritance.

## 2 Material and methods

### 2.1 Subjects

A total of 90 unrelated Brazilian individuals were selected for having hearing loss of presumptive autosomal recessive inheritance. They were referred by the following institutions: DERDIC (Divisão de Educação e Reabilitação de Distúrbios Auditivos da Comunicação da Pontifícia Universidade Católica), Hospital das Clínicas da Faculdade de Medicina da USP e UNIFESP (Universidade Federal de São Paulo), all in the state of São Paulo, and other institutions. They were ascertained between 2000 and 2019 at the Human Genome and Stem Cell Research Center, Instituto de Biociências, Universidade de São Paulo, São Paulo, SP, Brazil. They were selected based on pedigree criteria, being either born from consanguineous couples or belonging to families with two or more affected individuals in the sibship and most cases were of normal-hearing parents. Although most probands presented with non-syndromic hearing loss, individuals with suspected syndromic deafness were not excluded, as long as they followed at least one of the previous criteria. The study was approved by the Ethics Committee, Institute of Biosciences, University of São Paulo (1.133.416–23/06/2015). Written informed consent was obtained from all hearing-impaired individuals, and relatives, or their legal guardians. Information about secondary actionable findings was not offered to patients because samples were collected in research focused on hearing loss, and written informed consent authorized investigation of genetic factors related to hearing loss, exclusively.

In all probands, the molecular cause of hearing loss had been previously investigated by screening of the c.35delG at *GJB2* (NM_004004.5, GRC37) (Scott et al., 1998), screening of two *GJB6* deletions, del(GJB6-D13S1830) – chr13: 20,797,177–21,105,967:GRCh37 ∼309Kb, and del(GJB6-D13S1854) – chr13: 20,802,726–21,034,755:GRCh37 ∼232 Kb (Del Castillo et al., 2005), and screening of m.1555A>G at *MT-RNR1* (NC_012920.1) (Estivil et al., 1998). Most probands also had the coding region of *GJB2* previously analyzed through Sanger sequencing. Only individuals without an identified molecular cause for hearing loss after this screening were included. However, four cases identified with hypomorphic variants in *GJB2* were included in order to verify if there was another plausible genetic cause for their hearing loss.

### 2.2 DNA extraction

Blood samples were collected and DNA was extracted using different techniques available at the time of collection of samples: conventional phenol/chloroform extraction, commercial kits, or through the equipment Autopure LS (Gentra Systems, Minneapolis, United States) or QiaSimphony (Qiagen, Hilden, Germany).

### 2.3 Next-generation sequencing genes panel

The custom panel was designed using the SureDesign tool (https://earray.chem.agilent.com/suredesign/) and the DNA libraries were prepared according to the SureSelect QXT Target Enrichment System Kit for Illumina Multiplexed Sequencing (Agilent, Santa Clara, CA) protocol. Sequencing was performed on the HiSeq 2500 platform (Illumina, San Diego, California, United States). Sequences were aligned with the Burrows-Wheeler Aligner (BWA) (http://bio-bwa.sourceforge.net/). The Picard tool (http://broadinstitute.github.io/picard/) was used to eliminate PCR duplicates, identify indels, and realign reads. Recalibration of base qualities and variant calling were performed using Genome Analysis ToolKit (GATK) (https://github.com/broadinstitute/gatk) and the annotation of variants was performed using Annovar (http://annovar.openbioinformatics.org/en/latest/). The list of 99 genes is presented in Supplementary Material, in Supplementary Table S1.

### 2.4 Whole exome sequencing

Libraries were captured and constructed through one of the following kits: Nextera Rapid Capture (Illumina, San Diego, California, United States), TruSeq Exome Library Prep Kit (Illumina, San Diego, California, United States), SureSelectQXT Target Enrichment (Agilent Technologies, Santa Clara, California, United States) and xGen Exome Research Panel v1 (Integrated DNA Technologies, Coralville, Iowa, United States). Sequencing was performed on the HiSeq 2500 platform (Illumina, San Diego, California, United States). Bioinformatics procedures were the same as previously described for the next-generation targeted sequencing of 99 genes.

### 2.5 Variant filtering, interpretation, and classification

Exonic and splicing variants were filtered considering their minor allele frequency under 1% in the following databases: Exome Aggregation Consortium (ExAC, exac.broadinstitute.org), NHLBI Exome Sequencing Project (ESP, http://evs.gs.washington.edu/EVS/), 1000 Genomes Project (www.internationalgenome.org), and the Brazilian ABraOM (http://abraom.ib.usp.br). The pathogenicity of the candidate variants was predicted through REVEL, CADD Phred, and for splicing variants through MAX EntScan, ADA Score, RF Score, and SpliceAI. Pathogenicity information was checked at ClinVar (https://www.ncbi.nlm.nih.gov/clinvar/) and Deafness Variation Database (http://deafnessvariationdatabase.org/). The GRC37 assembly was used as a reference.

The whole exome sequencing analysis focused on variants present in genes previously associated with hearing loss included at “The Hereditary Hearing Loss Homepage” (https://hereditaryhearingloss.org/), Online Mendelian Inheritance in Man (OMIM, https://www.omim.org/phenotypicSeriesTitles/all) or “Deafness Variation Database”.

Candidate variants were classified according to the American College of Medical Genetics and Genomics (ACMG) and Association for Molecular Pathology (AMP) guidelines for genetic hearing loss (Richards et al., 2015; Oza et al., 2018).

### 2.6 Sanger sequencing

Sanger sequencing was used to confirm the presence of candidate variants and, whenever DNA samples of family members were available, to determine segregation and trans configuration. This information was used in the ACMG classification. Primers to amplify exons containing candidate variants were designed using Primer-BLAST (https://www.ncbi.nlm.nih.gov/tools/primer-blast/). PCR products were purified using Exonuclease I (Affymetrix, Santa Clara, California, United States) and Shrimp Alkaline Phosphatase (Affymetrix, Santa Clara, California, United States). The purified products were prepared using BigDye terminator v3.1 (Thermo Fisher Scientific, Waltham, Massachusetts, United States) and analyzed with the 48-capillary ABI 3730 DNA Analyser (Thermo Fisher Scientific, Waltham, Massachusetts, United States).

## 3 Results

### 3.1 Cohort characterization

**FIGURE 1:**
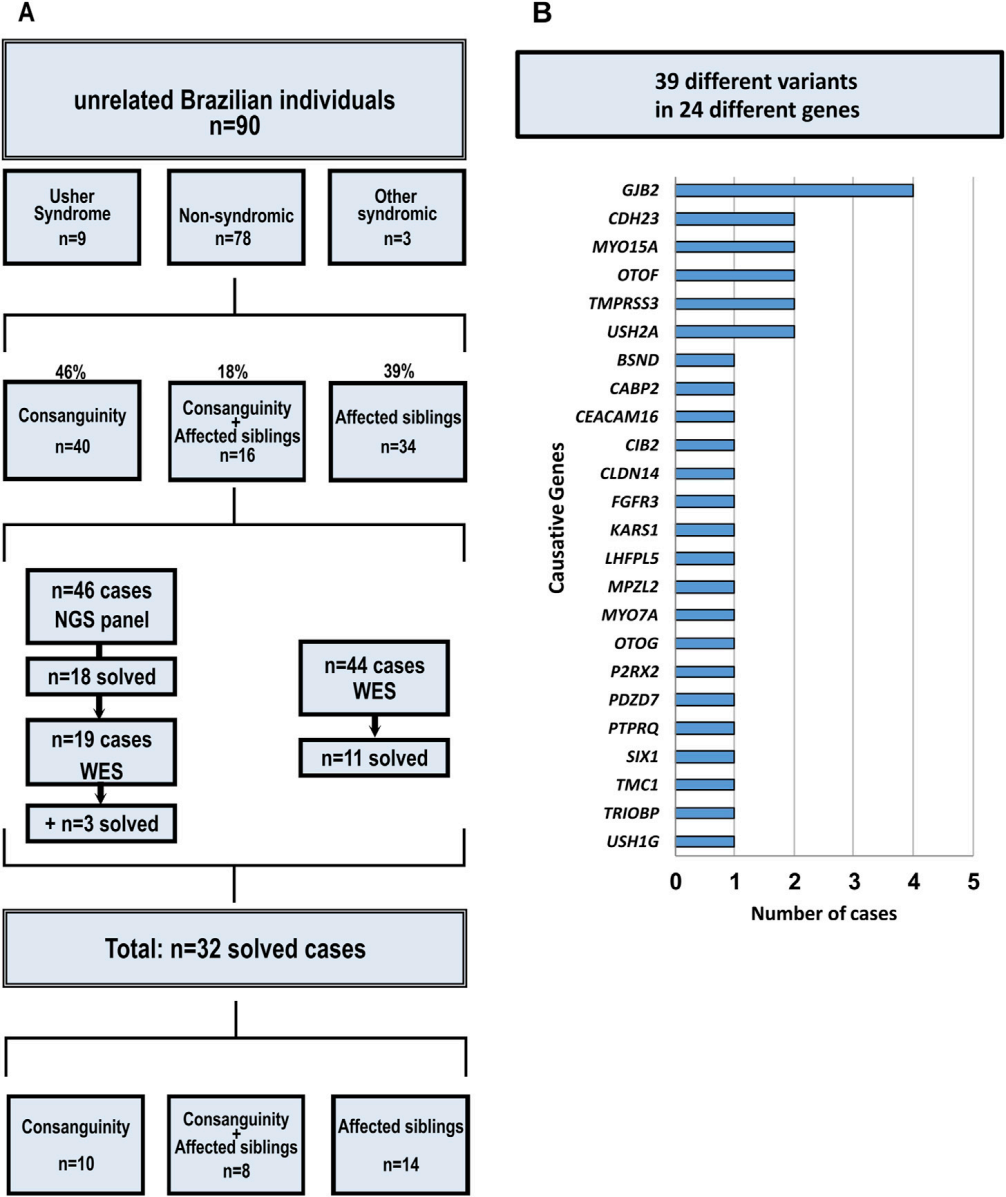
Cohort description and contribution of genes. **(A)** Cohort Characterization and Strategies; **(B)** Contribution of each gene to the solved cases.

Among the 90 probands, 38% (34/90) were from families with two or more affected individuals in the sibship, 44% (40/90) were born from consanguineous couples, and 18% (16/90) fulfilled both criteria. Nine of the 90 probands had been previously clinically diagnosed with Usher syndrome (Figure 1A).

Forty-six probands had their genetic material analyzed through next-generation targeted sequencing of 99 hearing loss-related genes (Supplementary Material). Due to the discovery of new genes associated with hearing loss that resulted in the need to update repeatedly the customized panel, 63 probands had their genetic material analyzed through whole exome sequencing: 19 probands without identified causative variants identified after the next-generation targeted sequencing of 99 genes panel and 44 additional probands, that had not been previously studied through NGS technologies.

In 32 of the 90 probands (36%) the causative variants were identified: 18 probands after next-generation targeted sequencing of 99 genes, 11 probands after whole exome sequencing and three cases whose causative variants were found after both (Tables 1, 2; Figure 1A). Pedigrees and a summary of the investigation of segregation in families are shown in Supplementary Figure S1. Tables 1, 2 and Figure 1A summarize the identified variants through all the strategies.

**TABLE 1:** Description of the probands phenotype and the causative variants identified.

Proband ID	Family history	Clnical Data	NGS method	Gene	*Genomic Position:GRC37-hg19*	OMIM	Ref Seq	DNA	Protein
RC-01	Consanguinity, 2 affected siblings	Postlingual progressive	Panel	*CEACAM16*	*19:45207341:C>T*	DFNB113	NM_001039213.1	**c.436C>T**	**p.(Arg146Ter)**
						(#618410)			
RC-02	Consanguinity, affected brother	Congenital profound	Panel	*CLDN14*	*21:37833703:G>T*	DFNB29	NM_144492.2	c.291C>A	p.(Cys97Ter)
						(#614035)			
RC-03	3 affected brothers, 1 affected sister	Postlingual (11-12y) progressive	Panel	*GJB2*	13:20763620:A>G	DFNB1A	NM_004004.5	c.101T>C	p.(Met34Thr)
					13:20763171:G>A	(#220290)		c.550C>T	p.(Arg184Trp)
RC-04	Affected sister	Prelingual profound	Panel	*MYO7A*	11:76909587:G>C	DFNB2	NM_000260.3	c.4489G>C	p.(Gly1497Arg)
						(#600060)			
RC-05	Consanguinity, affected brother	Prelingual progressive	Panel	*TMPRSS3*	*21:43803278:G>A*	DFNB8	NM_032405.1	c.646C>T	p.(Arg216Cys)
						(#601072)			
RC-06	Consanguinity	Postlingual mild	Panel	*GJB2*	13:20763612:C>T	DFNB1A	NM_004004.5	c.109G>A	p.(Val37Ile)
						(#220290)			
RC-07	Consanguinity, affected father	Postlingual	Panel	*SIX1*	*14:61115522:T>C*	Branchiootic syndrome 3	NM_005982.3	c.386A>G	p.(Tyr129Cys)
						(#608389)			
RC-08	Affected sister	Postlingual progressive moderate	Panel	*GJB2*	13:20763620:A>G	DFNB1A	NM_004004.5	c.101T>C	p.(Met34Thr)
						(#220290)			
RC-09	Consanguinity	Postlingual (17y) progressive moderate	Panel	*GJB2*	13:20763620:A>G	DFNB1A	NM_004004.5	c.101T>C	p.(Met34Thr)
						(#220290)			
RC-10	Affected sister	Prelingual profound	Panel	*OTOF*	*2:26698882:G>T*	DFNB9	NM_194248.2	c.2891C>A	p.(Ala964Glu)
					*2:26688592:G>A*	(#601071)		c.4747C>T	p.(Arg1583Cys)
RC-11	Consanguinity, affected brother	Usher syndrome: prelingual,RP detected in the third decade	Panel	*USH1G*	*17:72916189:G>A*	Usher syndrome type 1G	NM_001282489.1	c.433C>T	p.(Gln145Ter)
						(#606943)			
RC-12	Affected sister	Prelingual profound	Panel	*LHFPL5*	6:35782436:C>T	DFNB67	NM_182548.3	c.526C>T	p.(Arg176Cys)
RC-13	Consanguinity, affected brother	Usher syndrome: prelingual moderate to severe, RP and low sperm count	Panel	*USH2A*	*1:216011357:GGT>G*	Usher syndrome type 2ª	NM_206933.2	c.9345_9346del	p.(Pro3116Hisfs*13)
						(#276901)			
RC-14	Consanguinity, 2 affected sisters	N/A	Panel	*PTPRQ*	*12:80862506:C>CAT*	DFNB84	NM_001145026.2	**c.473_474insTA**	p.(Gln158Hisfs*5)
						(#613391)			
RC-15	Consanguinity	Usher syndrome, prelingual profound, RP in the first decade	Panel	*CDH23*	*10:73442222:CT>C*	Usher syndrome type 1C	NM_022124.5	**c.1880delT**	p.(Leu627Argfs*10)
						(#601067)			
RC-16	Affected sister	Congenital profound	Panel	*CDH23*	*10:73491848:G>A*	DFNB12	NM_022124.5	c.3820G>A	p.(Glu1274Lys)
					*10:73565593:G>T*	(#601386)		c.7903G>T	p.(Val2635Phe)
RC-17	Affected sister	Prelingual profound	Panel	*TMC1*	*9:75309631:G>A*	DFNB7	NM_138691.2	c.236+1G>A	Splicing
					*9:75404103:G>A*	(#600974)		**c.1094G>A**	**p.(Arg365Lys)**
RC-19	3 affected siblings	Usher syndrome: postlingual bilateral, RP onset 13y	Panel	*USH2A*	*1:215853685:C>A*	Usher syndrome type 2A	NM_206933.2	**c.12100G>T**	**p.(Glu4034Ter)**
					*1:215848036:A>G*	(#276901)		c.13217T>C	p.(Leu4406Pro)
RC-20	Affected sisters	Auditory neuropathy	WES	*OTOF*	*2:26688904:T>C*	DFNB9	NM_194248.2	**c.4541A>G**	**p.(Asp1514Gly)**
					*2:26687737:C>T*	(#601071)		c.4960G>A	p.(Gly1654Ser)
RC-21	Consanguinity	Perilingual progressive moderate	WES	*CABP2*	*11:67288601:G>A*	DFNB93	NM_001318496.2	c.292C>T	p.(Arg98Ter)
						(#607314)			
RC-22	Consanguinity	Prelingual prfound	WES	*MYO15A*	*17:18062662:G>A*	DFNB3	NM_016239.3	c.9229+1G>A	Splicing
						(#602666)			
RC-23	Consanguinity	Profound	WES	*BSND*	*1:55464998:G>A*	Bartter syndrome, type 4A	NM_057176.3	c.139G>A	p.(Gly47Arg)
						(#602522)			
RC-24	Affected brother	N/A	WES	*MYO15A*	*17:18035812:G>A*	DFNB3	NM_016239.4	c.4252G>A	p.(Gly1418Arg)
					*17:18057202:C>A*	(#602666)		c.8080C>A	p.(Arg2694Ser)
RC-25	Consanguinity	Postlingual (first decade) mixed progressive, cataract due to the *DNMBP* gene	Panel, WES	*P2RX2*	*12:133195521:G>GC*	DFNA41	NM_170682.4	**c.121dup**	p.(Leu41Profs*231)
						(#600844)			
RC-26	Consanguinity	Prelingual mild to moderate, camptodactyly, microcephaly, flexion restriction of the third digit	Panel,WES	*FGFR3*	*4:1807369:A>G*	CATSHL syndrome (#610474)	NM_000142.4	c.1618A>G	p.(Asn540Asp)
RC-27	Affected brother	Prelingual stable severe, Marfan Syndrome	Panel, WES	*PDZD7*	*10:102789810:C>CG*	DFNB57	NM_001195263.2	c.166dup	p.(Arg56Profs*24)
					*10:102770434:GC>G*	(#612971)		c.2211del	p.(Gln737Hisfs*16)
RC-28	Consanguinity	Postlingual (first decade) stable profound	WES	*TRIOBP*	*22:38121858:C>T*	DFNB28	NM_001039141.2	c.3295C>T	p.(Gln1099Ter)
						(#609761)			
RC-29	Affected siblings	Postlingual (first decade) progressive moderate	WES	*OTOG*	*11:17575066:G>T*	DFNB18B	NM_001277269.2	**c.576G>T**	**p.(Gln192His)**
					*11:17615300:G>A*	(#604487)		**c.3321G>A**	**p.(Trp1107Ter)**
RC-30	Affected siblings	Postlingual progressive severe and ataxia	WES	*KARS1*	*16:75669880:G>A*	DFNB89 (#613916)	NM_001130089.2	c.683C>T	p.(Pro228Leu)
RC-31	Affected siblings	Postlingual (first decade) profound	WES	MPZL2	11:118133798:CT>C	DFNB111 (#618145)	NM_144765.2	c.72delA	p.(Ile24Metfs*22)
RC-32	Consanguinity, affected siblings	Congenital profound	WES	*CIB2*	*15:78397661:G>A*	DFNB48 (#609439)	NM_006383.3	c.556C>T	p.(Arg186Trp)
RC33	Consanguinity, affected siblings	Postlingual progressive profound sensorineural	WES	*TMPRSS3*	21:43803278:G>A	DFNB10 (#601072)	NM_032405.2	c.646C>T	p.(Arg216Cys)

Novel variants are in bold, N/A = absent in the database, P = pathogenic, LP = likely pathogenic, VUS = variant of uncertain significance. All cases were bilateral, and except for RC-15 all were sensorineural. Splice prediction tools = MAX EntScan/ADA, RF Score, Splice.

**TABLE 2:** Description of further details regarding the causative variants identified

Proband ID	Gene	Exon	DNA	Protein	Zygosity	dbSNP	ClinVar	DVD	Alle Freq gnomAD	Segregation analysed?	CADD Phred	REVEL	Splicing prediction tools	ACMG	Criteria
RC-01	*CEACAM16*	4	**c.436C>T**	**p.(Arg146Ter)**	Hom	rs1435499034	N/A	P	0,00000908	Dias et al. 2019	33	N/A	N/A	P	PVS1, PM2, PM3, PP1
RC-02	*CLDN14*	3	c.291C>A	p.(Cys97Ter)	Hom	rs767108790	N/A	P	0,0000176	Yes	29.7	N/A	N/A	P	PVS1, PM2, PM3, PP1, PP5
RC-03	*GJB2*	2	c.101T>C	p.(Met34Thr)	Het	rs35887622	P	P	0,00887	No	20.9	0.702	N/A	LP	PM1, PM2, PM3, PM5, PP2, PP3, PP5
		2	c.550C>T	p.(Arg184Trp)	Het	rs998045226	P	P	0,0000173		25.8	0.954	N/A	P	PS4, PM1, PM2, PM3, PM5, PP2, PP3
RC-04	*MYO7A*	34	c.4489G>C	p.(Gly1497Arg)	Hom	N/A	P	P	0	Yes	30.0	0.950	N/A	P	PS4, PM2, PM3, PM5, PP1, PP3 , PP5
RC-05	*TMPRSS3*	8	c.646C>T	p.(Arg216Cys)	Hom	rs145913750	N/A	P	0,0000259	Yes	28.6	0.833	N/A	P	PM1, PM2, PM3, PM5, PP1, PP3
RC-06	*GJB2*	2	c.109G>A	p.(Val37Ile)	Hom	rs72474224	P	P	0,00879	No	21.7	0.656	N/A	LP	PM1, PM2, PM3, PM5, PP3, PP5
RC-07	*SIX1*	1	c.386A>G	p.(Tyr129Cys)	Het	rs104894478	P	P	0,00000866	No	32	0.946	N/A	P	PS3, PS2, PM1, PM2, PM5, PP1, PP3, PP5
RC-08	*GJB2*	2	c.101T>C	p.(Met34Thr)	Hom	rs35887622	P	P	0,00887	No	20.9	0.702	N/A	LP	PM1, PM2, PM3, PM5, PP2,PP3, PP5
													N/A		
RC-09	*GJB2*	2	c.101T>C	p.(Met34Thr)	Hom	rs35887622	P	P	0,00887	No	20.9	0.702	N/A	LP	PM1, PM2, PM3, PM5, PP2,PP3, PP5
RC-10	*OTOF*	24	c.2891C>A	p.(Ala964Glu)	Het	rs201329629	P	P	0	Yes	33	0.371	N/A	LP	PM2, PM3, PP1, PP3, PP5
		38	c.4747C>T	p.(Arg1583Cys)	Het	rs781688103	P	P	0,000035		32	0.883	N/A	P	PM2, PM3, PM5, PP3, PP5
RC-11	*USH1G*	2	c.433C>T	p.(Gln145Ter)	Hom	rs773231689	N/A	VUS	0,0000274	No	40	N/A	N/A	P	PVS1, PM2, PM3
RC-12	*LHFPL5*	2	c.526C>T	p.(Arg176Cys)	Hom	rs768655651	N/A	VUS	0,0000147	Yes	31	0.804	N/A	LP	PM1, PM2, PM3, PM5, PP1, PP3
RC-13	*USH2A*	47	c.9345_9346del	p.(Pro3116Hisfs*13)	Hom	rs536593247	P/LP	P	0,0000606	Yes	N/A	N/A	N/A	P	PVS1, PM2, PM3, PP1 , PP5
RC-14	*PTPRQ*	9	**c.473_474insTA**	p.(Gln158Hisfs*5)	Hom	N/A	N/A	N/A	0	No	N/A	N/A	N/A	P	PVS1, PM2, PM3
RC-15	*CDH23*	18	**c.1880delT**	p.(Leu627Argfs*10)	Hom	N/A	N/A	N/A	0	Yes	N/A	N/A	N/A	P	PVS1, PM2, PM3, PP1
RC-16	*CDH23*	31	c.3820G>A	p.(Glu1274Lys)	Het	rs775540526	N/A	VUS	0,0000148	Yes	27.9	0.823	N/A	LP	PM2, PM3, PP3, PP1
		55	c.7903G>T	p.(Val2635Phe)	Het	rs763721044	VUS	P	0,0000265		27.6	0.590	N/A	LP	PM2, PP5, PP1, PP3
RC-17	*TMC1*	7	c.236+1G>A	Splicing	Het	rs775428246	P	P	0,0000315	Yes	N/A	N/A	native loss HIGH, 0.9999 (splice disrupting), 0.92 (donor loss)	P	PVS1, PM2, PM3, PP1 , PP5
		15	**c.1094G>A**	**p.(Arg365Lys)**	Het	rs1211839569	N/A	N/A	0		27.3	0.658	N/A	LP	PM2, PM3, PP3, PP1, PP2
RC-19	*USH2A*	62	**c.12100G>T**	**p.(Glu4034Ter)**	Het	N/A	N/A	N/A	0	No	45	N/A	N/A	P	PVS1, PM2, PM3, PP5
		63	c.13217T>C	p.(Leu4406Pro)	Het	rs745693690	VUS	LP	0,0000173		21.4	0.338	N/A	VUS	PM2, PM3
RC-20	*OTOF*	37	**c.4541A>G**	**p.(Asp1514Gly)**	Het	N/A	N/A	N/A	0	Yes	28.7	0.923	N/A	LP	PM2, PM3, PP1, PP3
		39	c.4960G>A	p.(Gly1654Ser)	Het	rs1005694756	N/A	P	0,00000893		33	0.371	N/A	LP	PM2, PM3, PP1, PP3
RC-21	*CABP2*	4	c.292C>T	p.(Arg98Ter)	Hom	rs761766884	N/A	VUS	0,00000737	No	36	N/A	N/A	P	PVS1, PM2, PM3
RC-22	*MYO15A*	54	c.9229+1G>A	Splicing	Hom	N/A	N/A	P	0	No	N/A	N/A	native loss (HIGH), 0.9999 (splice disrupting), 0.99 (donor loss)	P	PVS1, PM2, PM3
RC-23	*BSND*	1	c.139G>A	p.(Gly47Arg)	Hom	rs74315289	P	P	0,000104	No	24.4	0.495	N/A	P	PVS1, PM2, PM3, PP5
RC-24	*MYO15A*	11	c.4252G>A	p.(Gly1418Arg)	Het	rs753790346	N/A	P	0,0000148	N/A	26.1	0.955	N/A	P	PM2, PM3, PP3
		43	c.8080C>A	p.(Arg2694Ser)	Het	rs371730430	N/A	VUS	0,0000264		25.2	0.843	N/A	LP	PM2, PM3, PP3
RC-25	*P2RX2*	1	**c.121dup**	p.(Leu41Profs*231)	Het	N/A	N/A	N/A	0	N/A	N/A	N/A	N/A	LP	PVS1, PM2
RC-26	*FGFR3*	12	c.1618A>G	p.(Asn540Asp)	Hom	rs1057519049	LP/VUS	VUS	0,00000869	No	26.7	0.860	N/A	P	PS4, PM2, PM1, PM5, PP3
RC-27	*PDZD7*	2	c.166dup	p.(Arg56Profs*24)	Het	rs587776894	P	P	0,0000799	Yes	N/A	N/A	N/A	P	PVS1, PM2, PM3, PP5
		15	c.2211del	p.(Gln737Hisfs*16)	Het	N/A	N/A	VUS	0,0000174		N/A	N/A	N/A	P	PVS1, PM2, PM3
RC-28	*TRIOBP*	16	c.3295C>T	p.(Gln1099Ter)	Hom	rs777561677	N/A	VUS	0,00000887	No	N/A	N/A	N/A	P	PVS1, PM2, PM3
RC-29	*OTOG*	5	**c.576G>T**	**p.(Gln192His)**	Het	rs1211225181	N/A	N/A	0	No	34	0.160	N/A	VUS	PM2, PM3, PP3
		26	**c.3321G>A**	**p.(Trp1107Ter)**	Het	rs979900875	N/A	N/A	0		47	N/A	N/A	LP	PVS1, PM2
RC-30	*KARS1*	6	c.683C>T	p.(Pro228Leu)	Hom	rs201650281	P/LP	LP	0,0001	Yes	24.1	0.537	N/A	P	PS3, PM2, PM3, PP1
RC-31	MPZL2	2	c.72delA	p.(Ile24Metfs*22)	Hom	rs752672077	P/LP	P	0,0008	Yes	N/A	N/A	N/A	P	PVS1, PM2, PM3
RC-32	*CIB2*	6	c.556C>T	p.(Arg186Trp)	Hom	rs370359511	P/LP	N/A	0,00003659	Yes	32	0.418	N/A	LP	PM2, PM3, PP1, PP5
RC33	*TMPRSS3*	8	c.646C>T	p.(Arg216Cys)	Hom	rs145913750	P	P	0,00002031	No	28.6	0.833	N/A	P	PM1, PM3, PM5, PP5

Novel variants are in bold, N/A, absent in the database; DVD, Deafness Variation Database; P, pathogenic; LP, likely pathogenic; VUS, variant of uncertain significance. All cases were bilateral, and except for RC-15 all were sensorineural. Splice prediction tools = MAX EntScan/ADA, RF Score, Splice.

### 3.2 Next-generation targeted sequencing of 99 genes

Among the 46 subjects analyzed through the customized panel, 18 cases were solved (39%), and four had previously been clinically diagnosed with Usher syndrome.

The genetic cause of hearing loss in one proband was found to be a variant associated with autosomal dominant syndromic hearing loss (*SIX1*). In total, 24% (6/25) of the disease-causing variants were novel, being the variant c.436C>T in *CEACAM16* first described in Dias et al. (2019).

### 3.3 Whole exome sequencing

In 14 of the 63 probands (22%) in this group, the causative variants were identified. In three of these probands, no causative variants had been identified previously with the next-generation targeted sequencing of 99 genes: in one of them, the gene harboring the causative variant was not included in the customized panel (*FGFR3*). In the other two cases (*P2RX2* and *PDZD7*), the variants were not detected after targeted sequencing probably because of technical issues. Causative variants related to syndromic hearing loss were found in two probands (*BSND* and *FGFR3*). A causative variant leading to nonsyndromic autosomal dominant hearing loss was found in one proband (*P2RX2*). In total, 29% (4/14) of the disease-causing variants were novel.

## 4 Discussion

The detection of causative variants in autosomal recessive hearing loss is challenging due to some factors such as the extreme locus heterogeneity and the high amount of non-recurrent variants. Our study confirms the wide heterogeneity of ARHL, since causative variants were found in 24 different known hearing loss genes and, besides causative variants in *GJB2*, there was not a single recurrent pathogenic variant causing hearing loss in this sample. Besides, 10 out of the 39 causative variants in known hearing loss genes are novel (Table 1, 2, indicated in bold).

### 4.1 Characterization of cases with causative variants in genes previously related to syndromic hearing loss

Causative variants in genes described as leading to syndromic hearing loss were found in three probands (RC-26, RC-23, and RC-07) born from consanguineous parents.

Proband RC-26 had a likely pathogenic variant in homozygosis in *FGFR3* (NM_000142.4:.c.1618A>G:p.(Asn540Asp). The variant had been described with controversial status, but *FGFR3* was already associated with camptodactyly, tall stature, and hearing loss syndrome (CATSHLS). In the first evaluation, at 5 months old, the proband had only camptodactyly in one digit. At the age of 11 years and 2 months, microcephaly was observed. Height was average and flexion restriction of the third digit was observed in both hands. Information about phenotypic variability is limited in CATSHLS syndrome since only four families have been reported (Toydemir et al., 2006; Makrythanasis et al., 2014; Escobar et al., 2016; Cannova et al., 2024). However, the presence of camptodactyly associated with hearing loss and a likely pathogenic variant in *FGFR3* pointed to a phenotype related to the CATSHL syndrome spectrum. In three reported cases so far, heterozygous missense variants c.1861C>T:p.(Arg621Cys), and c.1637C>A:p.(Arg621His) were detected and dominant transmission was observed. In a fourth case, c.1637C>A:p.(Thr546Lys) in homozygosis was found in two brothers (Makrythanasis et al., 2014). The catalytic loop of the tyrosine kinase domain of the *FGFR3* gene is affected in all cases. The aminoacid residue affected by the variant here described, 540, is very close to the p.(Thr546Lys) variant and interestingly, autosomal recessive inheritance was observed (Makrythanasis et al., 2014), as in the present case. Substitutions of residue 540 were previously associated with hypochondroplasia, but the resulting aminoacid is lysin, p.(Asn540Lys), and not Asp, as in the present case (MIM * 134934). According to UniProt, all variants described above are related to residues located within the protein kinase domain (472–761).

Proband RC-23 presented a pathogenic variant in homozygosis, c.139G>A: p.(Gly47Arg) in *BSND*, previously associated with Bartter syndrome, type 4A. She was first evaluated at the Genetics Unit at the age of 2 years and 8 months, the second child of consanguineous and healthy parents, presenting failure to thrive and speech delay. She was born at term (37 weeks), with a birth weight of 2,240 g and length of 45 cm, after a pregnancy with no history of polyhydramnios. The mother denies vomiting and the child had an appropriate food intake. No facial dysmorphisms were retrieved. Audiologic evaluation revealed profound hearing loss. In the investigation for her failure to thrive, biochemical analysis revealed metabolic alkalosis (venous blood pH: 7.46, plasma bicarbonate: 30 mmol/L and hypokalemia (2.4mEq/L - NV:3.7–5.0). Other electrolyte abnormalities included hypochloremia (94mEq/L - NV: 97–107), hypocalcemia (1.08 mmol/L - NV:1.12–1.32), as well as increased urine choride/creatinine ratio (390.9mEq/gcreatinine - NV:39–348). She has been referred to the nephrology unit and has been followed with a diagnosis of Bartter syndrome. At the last evaluation, at the age of 8 years and 10 months, she was taking oral potassium supplementation and attending a special school, learning sign language. There was a catch-up on weight gain and growth: W: 22.7 Kg (p.10^th^-25^th^ centile) and H: 120 cm (p.5^th^-10^th^ centile). This syndrome exhibits great phenotypic variability (Bartter et al., 1962; Landau et al., 1995). When first ascertained by us, there was no report of kidney problems. The proband, after a recent reassessment, received a clinical diagnosis of Bartter syndrome due to defects in renal reabsorption associated with hearing loss, which is common in Bartter syndrome type 4A.

Proband RC-07 had a heterozygous pathogenic variant c.386A>G: p.(Tyr129Cys) in *SIX1* previously associated with Branchiootic syndrome type 3, which exhibits autosomal dominant pattern of inheritance. This syndrome also exhibits great phenotypic variability and hearing loss is the most frequent signal, found in more than 90% of the cases (SMITH, 2014). Clinical revaluation is needed, but it is possible that hearing loss is the only clinical finding in the proband.

This corroborates ACMG recommendations of periodic clinical revaluation since some phenotypic manifestations in syndromic forms appear later in life, after the onset of hearing loss (Alford et al., 2014), and, in some cases, clinical presentation is mild and early recognition of a syndrome may be difficult. These findings favor Whole Exome Sequencing as a first-tier exam in hearing loss, instead of customized panels, and depending on costs or feasibility, exclusion of DFNB1-related hearing loss could be done first, as we have done in our casuistic.

### 4.2 The relative contribution of different DFNB loci to ARHL

Despite the genetic heterogeneity, some genes showed greater contribution to cases of autosomal recessive deafness in our study: *GJB2*, *CDH23* (10.34%), *MYO15A* (7%), *OTOF* (7%), and *USH2A* (7%) (Figure 1B). It is important to note that the frequency of pathogenic variants in *GJB2* cannot be precisely calculated because it would be clearly underestimated, since many families with presumptive ARHL had been screened for variants in *GJB2* before this study. In fact, nearly 40 families with DFNB1-related pathogenic variants were known in our diagnostic settings before the sample selection for this study.

We confirmed with our data that the variants classified as “hypomorphic” in *GJB2*, such as c.101T>C: p.(Met34Thr) and c.109G>A:p.(Val37Ile) were in fact causative of the hearing loss, since no other relevant variants were detected, and the degree of hearing impairment is compatible with the occurrence of these variants.

When comparing data from different studies that investigated variants in known hearing loss genes in large cohorts, especially in probands from pedigrees with presumptive autosomal recessive inheritance, it is interesting to notice that, although there is some variation between different populations, some genes appear as the most frequently altered in more than one study. These genes include *MYO15A*, *MYO7A*, *SLC26A4*, *STRC*, and *USH2A* (Bademci et al., 2016; Sloan-Heggen et al., 2016; Yan et al., 2016; Zazo Seco et al., 2017; Morgan et al., 2018). It is important to notice that studies that considered *STRC* as frequently related to autosomal recessive hearing loss also searched for copy number variations. This analysis did not occur in a systematic way in our sample.

In an investigation performed in the city of Campinas, São Paulo, Brazil, 180 probands had 86 specific variants genotyped in known hearing loss genes through the MassARRAYiPLEX^®^ platform in 17 different genes. This study indicated that *GJB2*, *SLC26A4*, *MYO15A*, *OTOF*, and *CDH23* showed greater contribution in cases of autosomal recessive deafness (Svidnicki et al., 2015) However, this study screened only previously known variants and NGS, as performed in this study, allowed us to identify ten novel variants and solved additional cases.

In the study of Manzoli et al. (2016), conducted in the city of Monte Santo, Bahia, Brazil, 19 probands were selected for having hearing loss of presumptive autosomal recessive inheritance. They had their genetic material analyzed through next-generation targeted sequencing of 180 hearing loss-related genes. The hearing loss genes with causative variants detected in this group were *MYO15A* (x10) and *CLDN14* (x1). The variant c.4198G>A, p.(Val1400Met) in *MYO15A* was found in eight probands and was probably inherited from a common founder. This study focused on the importance of the p.(Val1400Met) variant in Northeastern Brazil. Other studies also indicated that, apart from *GJB2*, pathogenic variants in *SLC26A4*, *MYO15A*, *OTOF*, and *CDH23* are a frequent cause of hearing loss in European populations (Hilgert, Smith, Van Camp, 2009).

We did not detect variants in the *SLC26A4,* probably due to a previous study of this cohort (Nonose et al., 2018) that focused on linkage analysis and Sanger sequencing of this gene among candidate pedigrees.

### 4.3 Considerations about NGS as diagnostic tool in hearing loss

In the sample here presented, 32 of the 90 probands (36%) had the causative variants identified after NGS.

Next-generation sequencing clearly facilitated genetic testing for hearing loss. Targeted sequencing panels allow the identification of causative variants in known hearing loss genes and have been proven effective (Sloan-Heggen et al., 2016). It usually has better coverage and lower costs when compared to whole exome sequencing. However, custom panels need to be periodically updated due to the discovery of new genes, especially in heterogeneous conditions like hearing loss (Atik et al., 2015). In our study, for example, *FGFR3* was not included in the custom panel. Therefore, proband RC-26 had a negative result after next-generation targeted sequencing of 99 genes and the causative variant was detected only after whole exome sequencing. It is possible that patients with syndromic hearing loss who present mild clinical features of a syndrome may have been misclassified as “non-syndromic” in some studies.

A limitation of our study was the fact that not all probands were fully screened for copy-number-variants, which is an interesting aspect to investigate, for instance, with arrays or low-pass-whole genome sequencing (Mazzonetto et al., 2024).

Whole exome sequencing also provides the potential for the discovery of novel hearing loss genes, which means that we can focus on unsolved cases to search for candidate variants in genes that were not previously associated with hearing loss.

## 5 Conclusion

In conclusion, next-generation sequencing is a highly effective tool for the identification of causative variants in hereditary hearing loss, since it is an extremely genetic heterogeneous condition. To our knowledge, ours is the largest report of next-generation sequencing being applied to a cohort with autosomal recessive hearing loss in Brazil and in South America. In our study, the causative variants were identified in 32 of the 90 probands selected because of presumptive autosomal hearing loss (37%) and 28 probands (31%) had causative variants found in genes other than the *GJB2*. Both, custom panels and whole exome sequencing, exhibit advantages and disadvantages that must be evaluated before their application. We found a greater contribution of *CDH23*, *MYO15A*, *OTOF*, and *USH2A* in our cohort, besides the expected contribution of *GJB2.*

## Data Availability

The data presented in the study are deposited in the ClinVar repository (https://www.ncbi.nlm.nih.gov/clinvar/), accession number SUB14687443.
