# Supplementary material for: Genetic heterogeneity in autosomal recessive hearing loss: a survey of Brazilian families
Source: Front Genet. 2024 Oct 21;15:1409306. doi: 10.3389/fgene.2024.1409306 (PMC11532063; doi:10.3389/fgene.2024.1409306)
Supplement: Supplementary file 1 [file Image1.pdf]

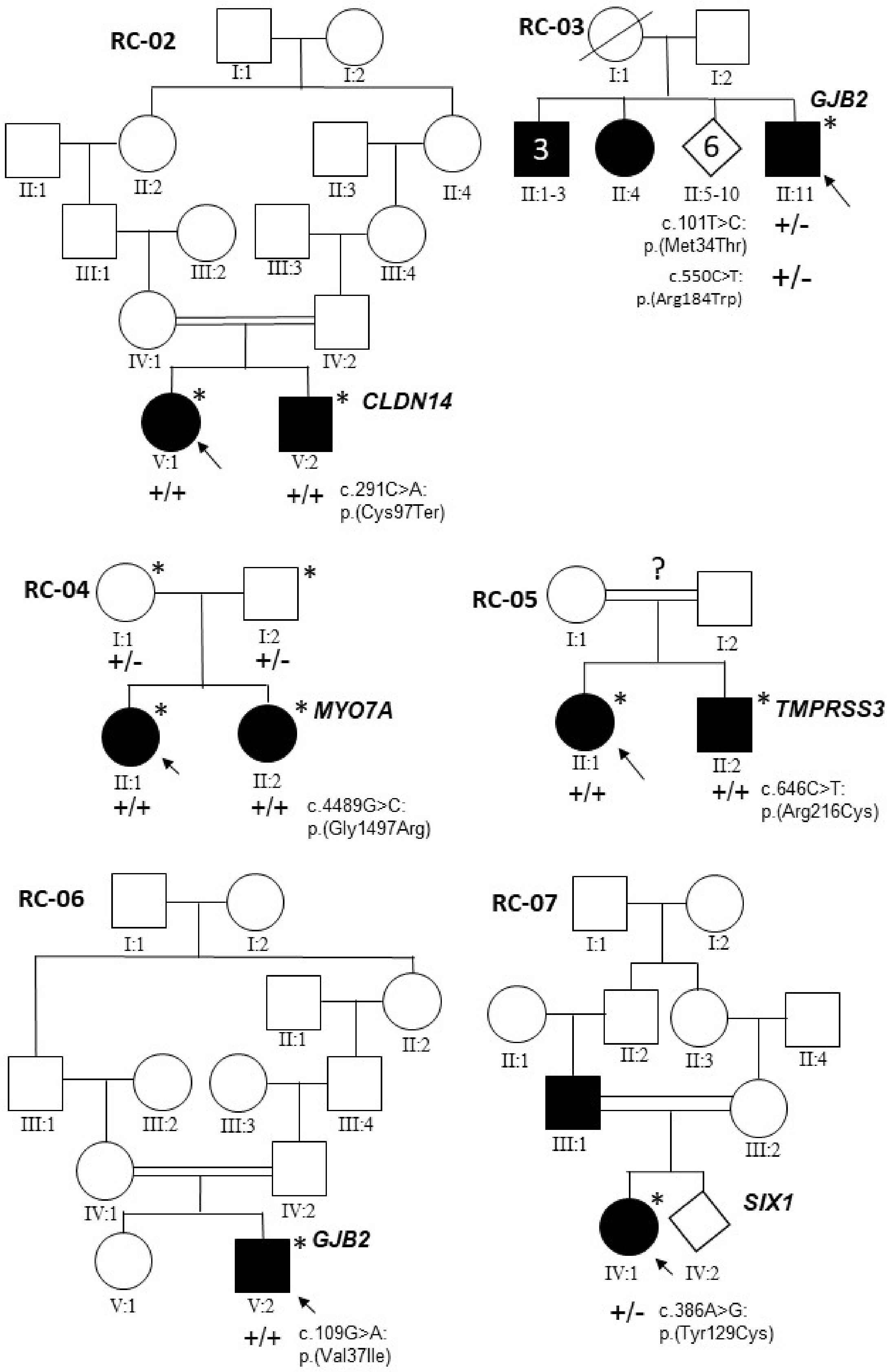

RC-02
I:1
I:2
II:1
II:2
II:3
II:4
III:1
III:2
III:3
III:4
IV:1
IV:2
V:1
V:2
*
* CLDN14
+/+
+/+
c.291C>A:
p.(Cys97Ter)
RC-03
I:1
I:2
GJB2
*
3
6
II:1-3
II:4
II:5-10
II:11
c.101T>C:
p.(Met34Thr)
+/-
c.550C>T:
p.(Arg184Trp)
+/-
RC-04
*
*
I:1
I:2
+/-
+/-
*
* MYO7A
II:1
II:2
+/+
+/+
c.4489G>C:
p.(Gly1497Arg)
RC-05
?
I:1
I:2
*
* TMPRSS3
II:1
II:2
+/+
+/+
c.646C>T:
p.(Arg216Cys)
RC-06
I:1
I:2
II:1
II:2
III:1
III:2
III:3
III:4
IV:1
IV:2
* GJB2
V:1
V:2
+/+
c.109G>A:
p.(Val37Ile)
RC-07
I:1
I:2
II:1
II:2
II:3
II:4
III:1
III:2
*
SIX1
IV:1
IV:2
+/-
c.386A>G:
p.(Tyr129Cys)

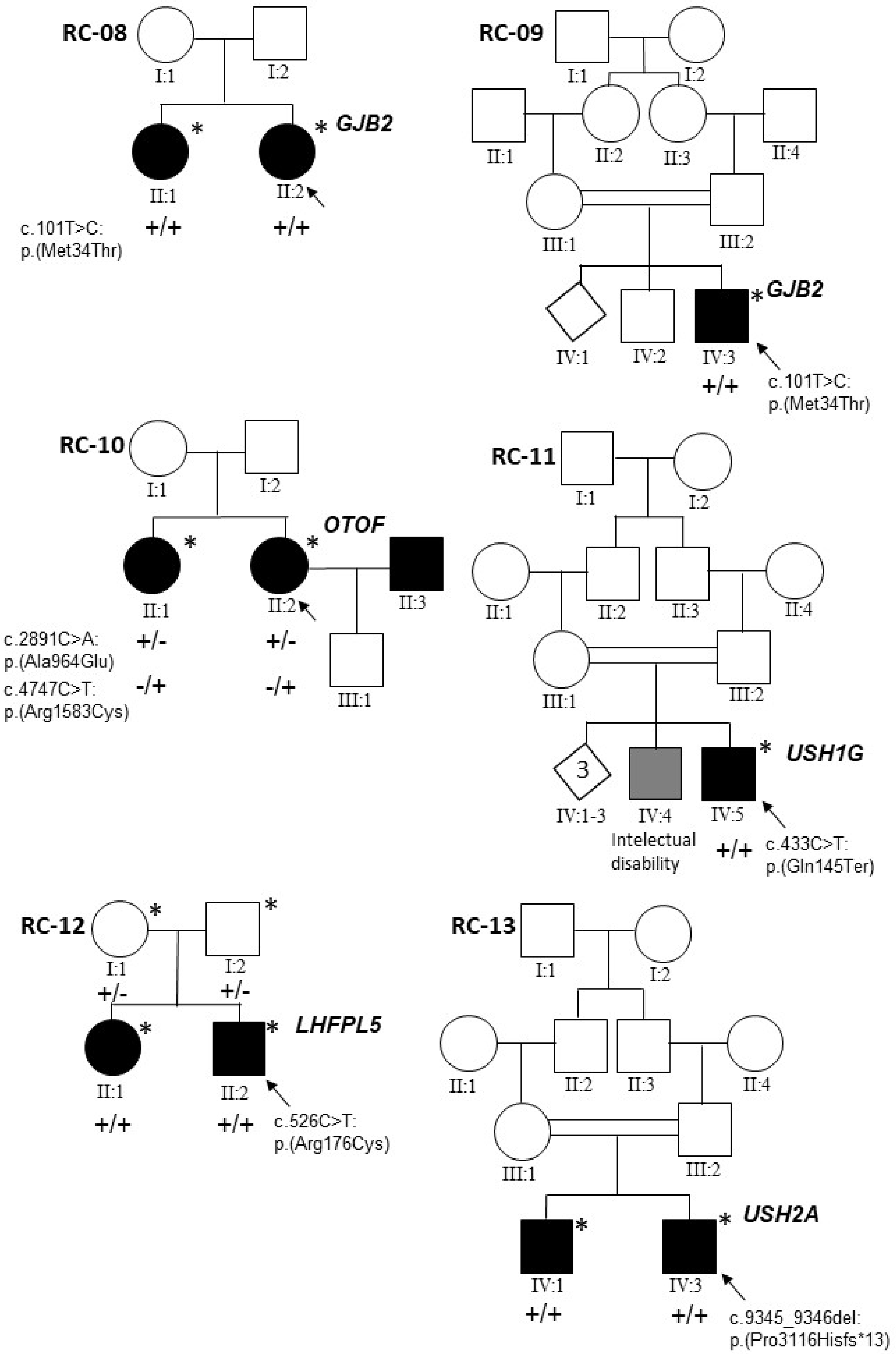

RC-08
I:1
I:2
GJB2
II:1
II:2
c.101T>C:
p.(Met34Thr)
+/+
+/+
RC-09
I:1
I:2
II:1
II:2
II:3
II:4
III:1
III:2
GJB2
IV:1
IV:2
IV:3
+/+
c.101T>C:
p.(Met34Thr)
RC-10
I:1
I:2
OTOF
II:1
II:2
II:3
c.2891C>A:
p.(Ala964Glu)
+/-
+/-
c.4747C>T:
p.(Arg1583Cys)
-/+
-/+
III:1
RC-11
I:1
I:2
II:1
II:2
II:3
II:4
III:1
III:2
USH1G
3
IV:1-3
IV:4
Intelectual
disability
IV:5
+/+
c.433C>T:
p.(Gln145Ter)
RC-12
I:1
+/-
I:2
+/-
LHFPL5
II:1
II:2
+/+
+/+
c.526C>T:
p.(Arg176Cys)
RC-13
I:1
I:2
II:1
II:2
II:3
II:4
III:1
III:2
USH2A
IV:1
IV:3
+/+
+/+
c.9345_9346del:
p.(Pro3116Hisfs*13)

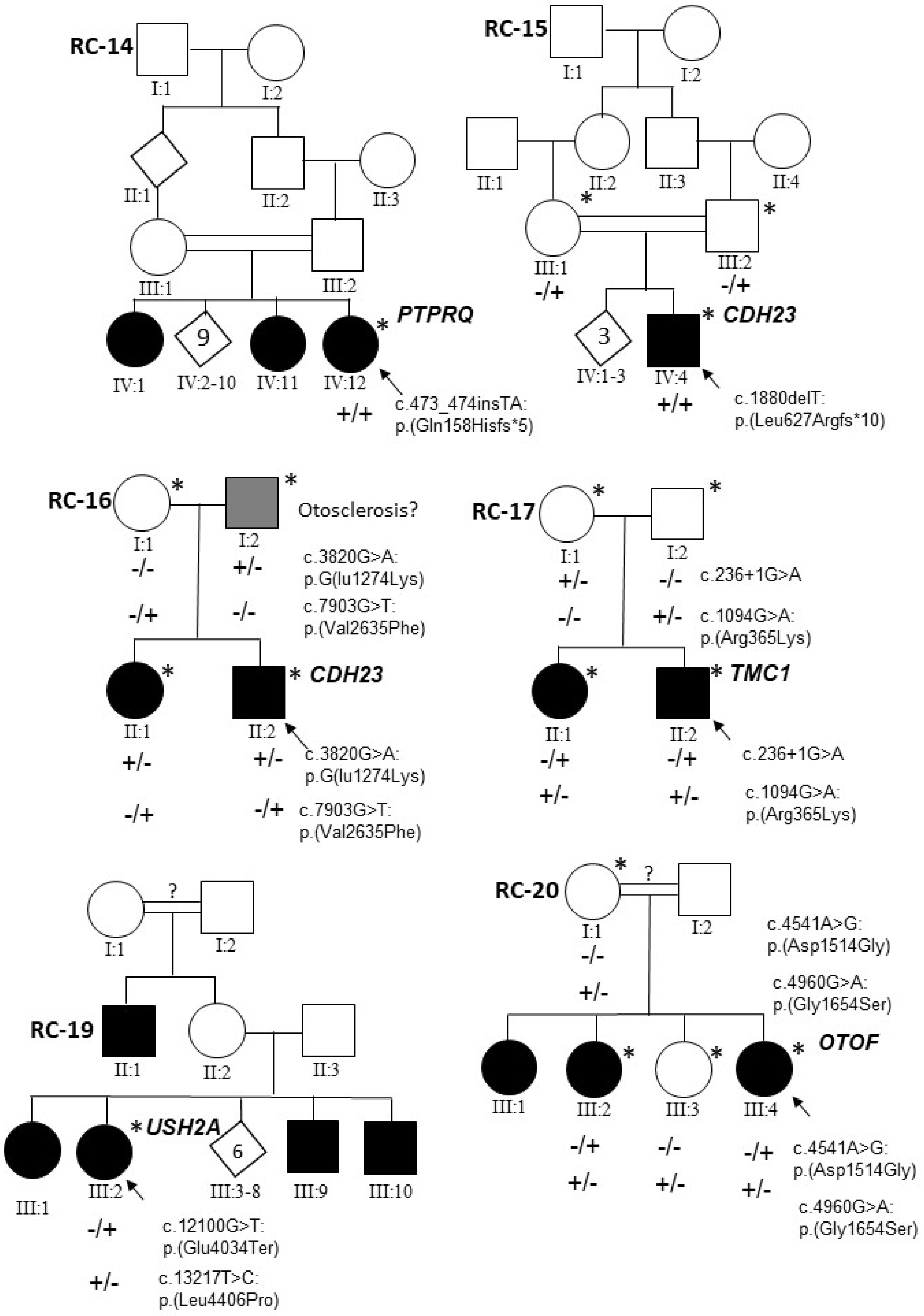

RC-14
I:1
I:2
II:1
II:2
II:3
III:1
III:2
9
IV:1
IV:2-10
IV:11
IV:12
PTPRQ
+/+
c.473_474insTA:
p.(Gln158Hisfs*5)
RC-15
I:1
I:2
II:1
II:2
II:3
II:4
III:1
-/+
III:2
-/+
3
IV:1-3
IV:4
+/+
CDH23
c.1880delT:
p.(Leu627Argfs*10)
RC-16
I:1
-/-
-/+
I:2
+/-
-/-
Otosclerosis?
c.3820G>A:
p.G(lu1274Lys)
c.7903G>T:
p.(Val2635Phe)
II:1
+/-
-/+
II:2
+/-
-/+
CDH23
c.3820G>A:
p.G(lu1274Lys)
c.7903G>T:
p.(Val2635Phe)
RC-17
I:1
+/-
-/-
I:2
-/-
+/-
c.236+1G>A
c.1094G>A:
p.(Arg365Lys)
II:1
-/+
+/-
II:2
-/+
+/-
TMC1
c.236+1G>A
c.1094G>A:
p.(Arg365Lys)
?
I:1
I:2
RC-19
II:1
II:2
II:3
6
III:1
III:2
III:3-8
III:9
III:10
USH2A
-/+
+/-
c.12100G>T:
p.(Glu4034Ter)
c.13217T>C:
p.(Leu4406Pro)
RC-20
?
I:1
-/-
+/-
I:2
c.4541A>G:
p.(Asp1514Gly)
c.4960G>A:
p.(Gly1654Ser)
III:1
III:2
-/+
+/-
III:3
-/-
+/-
III:4
-/+
+/-
OTOF
c.4541A>G:
p.(Asp1514Gly)
c.4960G>A:
p.(Gly1654Ser)

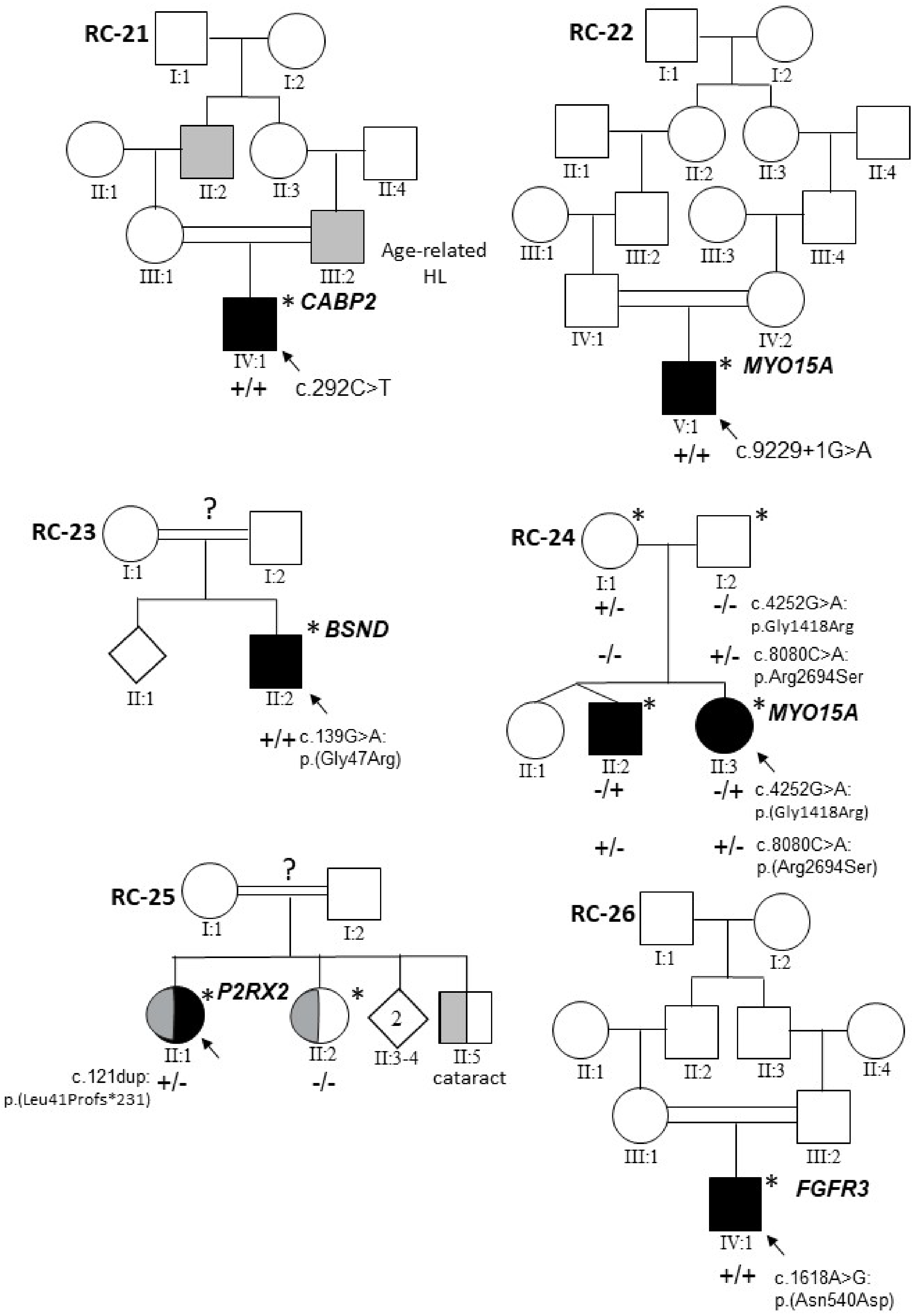
RC-21
I:1
I:2
II:1
II:2
II:3
II:4
III:1
III:2
Age-related HL
* CABP2
IV:1
+/+
c.292C>T
RC-22
I:1
I:2
II:1
II:2
II:3
II:4
III:1
III:2
III:3
III:4
IV:1
IV:2
* MYO15A
V:1
+/+
c.9229+1G>A
RC-23
?
I:1
I:2
II:1
* BSND
II:2
+/+ c.139G>A: p.(Gly47Arg)
RC-24
I:1
I:2
+/-
-/- c.4252G>A: p.Gly1418Arg
-/-
+/- c.8080C>A: p.Arg2694Ser
* MYO15A
II:1
II:2
II:3
-/+
-/+ c.4252G>A: p.(Gly1418Arg)
+/-
+/- c.8080C>A: p.(Arg2694Ser)
RC-25
?
I:1
I:2
* P2RX2
II:1
c.121dup: p.(Leu41Profs*231) +/-
II:2
-/-
2
II:3-4
II:5
cataract
RC-26
I:1
I:2
II:1
II:2
II:3
II:4
III:1
III:2
* FGFR3
IV:1
+/+
c.1618A>G: p.(Asn540Asp)

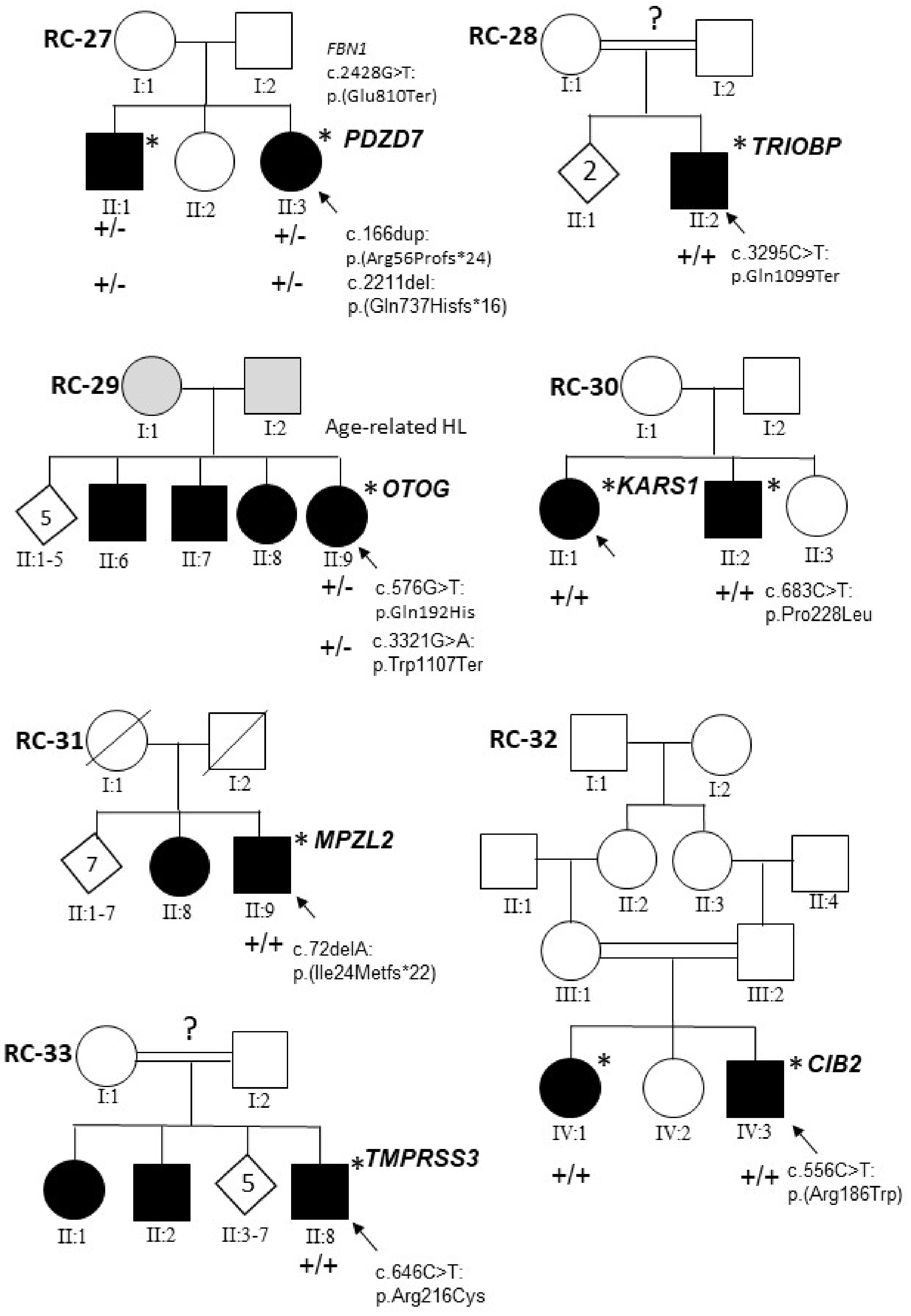
RC-27
I:1
I:2
FBN1
c.2428G>T:
p.(Glu810Ter)
* PDZD7
II:1
II:2
II:3
+/-
+/-
c.166dup:
p.(Arg56Profs*24)
+/-
+/-
c.2211del:
p.(Gln737Hisfs*16)
RC-28
?
I:1
I:2
* TRIOBP
2
II:1
II:2
+/+
c.3295C>T:
p.Gln1099Ter
RC-29
I:1
I:2
Age-related HL
* OTOG
5
II:1-5
II:6
II:7
II:8
II:9
+/-
c.576G>T:
p.Gln192His
+/-
c.3321G>A:
p.Trp1107Ter
RC-30
I:1
I:2
*KARS1
II:1
II:2
II:3
+/+
+/+
c.683C>T:
p.Pro228Leu
RC-31
I:1
I:2
* MPZL2
7
II:1-7
II:8
II:9
+/+
c.72delA:
p.(Ile24Metfs*22)
RC-32
I:1
I:2
II:1
II:2
II:3
II:4
III:1
III:2
* CIB2
IV:1
IV:2
IV:3
+/+
+/+
c.556C>T:
p.(Arg186Trp)
RC-33
?
I:1
I:2
*TMPRSS3
5
II:1
II:2
II:3-7
II:8
+/+
c.646C>T:
p.Arg216Cys

Supplementary Figure S1. Pedigrees of the families showing the segregation of the causative variants. Asterisks indicate the family members from whom DNA samples were obtained. Black symbols indicate the type of hearing loss described in Table 1. Gray symbols might have different meanings depending on the pedigree (indicated in each pedigree).
